# Hypoxia Dependent Inhibition of Glioblastoma Cell Proliferation, Invasion, and Metabolism by the Choline-Kinase Inhibitor JAS239

**DOI:** 10.3390/metabo15020076

**Published:** 2025-01-26

**Authors:** Claire Louise Kelly, Martyna Wydrzynska, Marie M. Phelan, Sofya Osharovich, Edward J. Delikatny, Violaine Sée, Harish Poptani

**Affiliations:** 1 Centre for Preclinical Imaging, Department of Molecular and Clinical Cancer Medicine, University of Liverpool, Liverpool L69 3BX, UK; claire-kelly@outlook.com; 2 Centre for Cell Imaging, Department of Biochemistry & Systems Biology, University of Liverpool, Liverpool L69 7BE, UK; martyna9706@gmail.com; 3 High Field NMR Facility, Department of Biochemistry & Systems Biology, University of Liverpool, Liverpool L69 7ZX, UK; marie.phelan@liverpool.ac.uk; 4 Department of Radiology, Perelman School of Medicine, University of Pennsylvania, Philadelphia, PA 19104, USA; sofyaosharo@gmail.com (S.O.); delikatn@pennmedicine.upenn.edu (E.J.D.)

**Keywords:** glioblastoma, metabolomics, metabolism, hypoxia, 3D spheroids, cell cycle, choline kinase inhibitor, cell tracking, cell invasion

## Abstract

**Background:** Elevated choline kinase alpha (ChoK) levels are observed in most solid tumors, including glioblastomas (GBM), and ChoK inhibitors have demonstrated limited efficacy in GBM models. Given that hypoxia is associated with resistance to GBM therapy, we hypothesized that tumor hypoxia could be responsible for the limited response. Therefore, we evaluated the effects of hypoxia on the function of JAS239, a potent ChoK inhibitor in four GBM cell lines. **Methods:** Rodent (F98 and 9L) and human (U-87 MG and U-251 MG) GBM cell lines were subjected to 72 h of hypoxic conditioning and treated with JAS239 for 24 h. NMR metabolomic measurements and analyses were performed to evaluate the signaling pathways involved. In addition, cell proliferation, cell cycle progression, and cell invasion parameters were measured in 2D cell monolayers as well as in 3D cell spheroids, with or without JAS239 treatment, in normoxic or hypoxic cells to assess the effect of hypoxia on JAS239 function. **Results:** Hypoxia and JAS239 treatment led to significant changes in the cellular metabolic pathways, specifically the phospholipid and glycolytic pathways, associated with a reduction in cell proliferation via induced cell cycle arrest. Interestingly, JAS239 also impaired GBM invasion. However, effects from JAS239 were variable depending on the cell line, reflecting the inherent heterogeneity of GBMs. **Conclusions:** Our findings indicate that JAS239 and hypoxia can deregulate cellular metabolism, inhibit cell proliferation, and alter cell invasion. These results may be useful for designing new therapeutic strategies based on ChoK inhibition, which can act on multiple pro-tumorigenic features.

## 1. Introduction

Primary glioblastoma (GBM) is the most common adult primary brain tumor, and the current standard of care is ineffective in curing the patient in most cases [1,2]. Survival ranges between 14 and 16 months after diagnosis, with a 5-year survival rate of less than 5%, making GBM the deadliest brain tumor in adults [3,4,5]. Currently, temozolomide, an alkylating agent, and bevacizumab, a vascular endothelial growth factor receptor inhibitor, are the only Food and Drug Administration (FDA)-approved systemic therapies for newly diagnosed and recurrent GBM, respectively [6,7,8]. Targeted therapy for GBM is yet to demonstrate a substantial survival benefit; therefore, there is an urgent need to develop novel therapies for these patients.

Molecular phenotypes of distinct regions within the same tumor can be diverse and can generate unique tumor microenvironments [9,10,11,12]. For example, tumor cells exposed to continuous low-oxygen (hypoxic) environments initiate specific genetic and molecular alterations mediated via the hypoxia-inducible transcription factor (HIF) [9,13]. This cellular adaptation response contributes to enhanced cell survival, resistance to therapies, and intratumoral heterogeneity [14]. The specific impact of prolonged hypoxia on drug efficacy, combined with intratumoral heterogeneity, generates variable tumor sensitivity to targeted therapies. Metabolic inhibitors can reduce therapy resistance by limiting the levels of critical metabolites necessary for DNA damage repair and by enhancing chemo- and radiotherapy sensitivity [15], thereby supporting the rationale for their use as potential adjuvants in chemotherapy.

Choline kinase (ChoK) is the first committed step in the Kennedy pathway for phosphatidylcholine synthesis, the most abundant phospholipid in eukaryotic cell membranes [16,17]. Inhibition of ChoK has shown promising results in in vivo models of breast cancer and, more recently in GBM [18,19,20]. However, given that hypoxia is associated with GBM therapy resistance, we hypothesized that tumor hypoxia could be a contributing factor to the limited therapeutic efficacy observed to date. Therefore, we evaluated the effect of hypoxia on the function of JAS239, a potent ChoK inhibitor, in four GBM cell lines. Due to the highly heterogeneous nature of GBMs, each cell line represents different aspects of human GBMs. For example, F98 and U-251 MG cells have mutant p53 and increased EGFR expression and recapitulate highly invasive and necrotic GBM tumors. U87-MG cells harbor mutations in the p14, p16, and PTEN genes and exhibit anaplastic features. These cell lines have been used in the assessment of many GBM therapies, including temozolomide and radiotherapy [21]. Moreover, when assessing the effects of hypoxia on drug efficiency in vitro, most studies use a standard 24 h pre-incubation hypoxic condition, which is too short to recapitulate the long-term cellular adaptation that occurs within hypoxic tumors.

Here, we investigated the metabolic changes and the effects on cell proliferation and invasion triggered by JAS239, as well as the impact of prolonged hypoxia (up to 96 h) on these mechanisms. We used 2D cell culture to investigate the JAS239 effects on cell proliferation, and 3D spheroid models to assess the effects on cell invasion, using the four different GBM cell lines of rat and human origin mentioned above.

## 2. Materials and Methods

### 2.1. Cell Lines

All GBM cell lines (F98, 9L, U-87 MG, and U-251 MG) were maintained at 37 °C in a 5% CO_2_ humidified atmosphere. The rat F98 (American Type Culture Collection [ATCC] CRL-2397, Manassas, VA, USA) and 9L LacZ (ATCC CRL-2200) GBM cells were cultured in Gibco Dulbecco’s Modified Eagle Medium high glucose (4.5 g/L) medium supplemented with 1% L-Glutamine and 10% fetal bovine serum (FBS). Human Uppsala 87 malignant glioma (U-87 MG; ATCC HTB-14 TM) and Uppsala 251 malignant glioma (U-251 MG; Cell Line Services CLS, GmbH, Eppelheim, Germany) cells were cultured in Gibco Minimum Essential Medium supplemented with 1% sodium pyruvate and 10% FBS. Cells were passaged every 2–3 days at 80–90% confluency until passage 10. For hypoxic preconditioning, the cells were incubated for 72 h (h) at 1% O_2_ (hypoxia) in a hypoxic workstation (Don Whitley Hypoxic Workstation, Bingley, West Yorkshire, UK) prior to drug treatment. After treatment, cells were incubated for an additional 24 h during drug exposure. Drug treatment was performed within the hypoxia workstation, without reoxygenation of the cells.

### 2.2. Stable Cell Lines

F98 and U-87 MG histone H2B monomeric red fluorescent protein (H2BmRFP), and F98 and 9L Luc Zs Green stable cell lines were generated for 3D spheroid experiments to enable cell tracking. A lentiviral transduction method was used to insert the pHIV-H2BmRFP plasmid (addgene plasmid number #18982. Addgene, Watertown, MA, USA) or pHIV-Luc-ZsGreen (addgene plasmid number #39196) plasmid. Briefly, 1.5 × 10^6^ HEK293T cells were seeded per 10 cm dish and incubated overnight at 37 °C. Cells were transfected with the lentiviral reagent, made up in a 4:2:1 ratio of vector:packaging:envelope. The plasmids were then transfected with polyethylenimine (PEI) at a ratio of 2:1 PEI:DNA in serum-free DMEM medium and incubated at 37 °C for a maximum of 16 h, followed by a media change. Three days after transfection, the conditioned media was collected, centrifuged at 1000× *g* for 5 min, and filtered through a 0.45 μM polyethersulfone filter. Virus-containing media was transferred to ultracentrifugation tubes, a 20% sucrose cushion was expelled beneath the media layer, and then ultracentrifugation was performed at 4 °C for 2 h at 21,000 rpm. Viral particles were concentrated in 600 μL PBS and applied to F98 and U-87 MG cells immediately. For transduction, 1.4 × 10^5^ F98 and U-87 MG cells were seeded into a T25 flask. The full 600 μL of concentrated virus was applied, and a media change was performed after 72 h.

### 2.3. Cell Viability

The measure of cellular metabolic activity was used as a surrogate to assess cellular viability. Metabolic activity was assessed using a 3-(4,5-Dimethylthiazol-2-yl)-2,5-diphenyltetrazolium bromide (MTT) assay. Cells were preincubated for 72 h at 21% O_2_ or 1% O_2_ at 37 °C and then treated with JAS239 using a half-log serial dilution from 100 µM. After treatment, cells were further incubated for 24 h in the same incubation conditions. Following treatment, 5 μL of 5 mg/mL MTT reagent (Sigma Aldrich, St. Louis, MO, USA) was added and incubated for 4 h. A total of 50 μL of 10% SDS /0.01 M HCl solubilizing reagent was added and incubated overnight at 21% O_2_/37 °C. Absorbance was read at 570 nm using a Spectramax plus384 (Molecular Devices, San Jose, CA, USA) plate reader. Background absorbance was subtracted from all conditions and plotted as a percentage of the DMSO control to determine the IC50 for each cell line.

### 2.4. NMR on Cell Extracts

Aqueous metabolites were extracted from cell samples, and 1D ^1^H NMR spectra were acquired.

#### 2.4.1. Acetonitrile Extraction

Aqueous metabolites were extracted using 400 μL of ice-cold solution of 50% HPLC-grade acetonitrile: 50% double distilled water per sample. Samples were sonicated in an ice bath (4 °C) for 3 × 30 s (sec) at 10 kHz using a microtip probe. Samples were then vortexed and centrifuged for 5 min/12,000× *g*/4 °C. The cell pellets were discarded, and the supernatants were snap-frozen in liquid nitrogen and lyophilized overnight. Samples were stored at −80 °C until further use.

#### 2.4.2. ^1^H NMR Sample Preparation and Data Acquisition

Samples were reconstituted in a solution containing sodium phosphate (pH 7.4), deuterated (d4) trimethyl silyl propionate (TSP), 1.2 mM sodium azide, and 99.8% ^2^H_2_O. Samples were vortexed for 30 s followed by centrifugation at 12,000× *g* for 5 min/4 °C. Samples were then transferred using glass Pasteur pipettes into NMR tubes. 1D ^1^H NMR spectra were acquired using a Bruker Avance III HD 700 MHz spectrometer (Bruker, Ettlingen, Germany) with a 5 mm TCI cryoprobe at pH 7.4/25 °C. 1D 1H spectra were acquired for each sample along with water suppression, using the Bruker pulse sequence cpmgpr1d (Carr–Purcell–Meiboom–Gill [CPMG]) with 256 transients, a 15 ppm spectral width, 32K points, and a 3.1 sec acquisition time. Full experimental parameters and pulse sequences are available with the deposited data at https://www.ebi.ac.uk/metabolights/editor/MTBLS6212/descriptors (metabolights ID MTBLS 6212).

#### 2.4.3. Spectral Processing and Data Analysis

Initial processing of each spectrum was carried out in Bruker Topspin v3.2. Chenomx v8.2 software (www.chenomx.com, Edmonton, Canada) was used for metabolite annotation with identification against the in-house metabolite library. The spectra were normalized to the total NMR signal intensity from each sample, and integrals bucketed per peak into metabolite-specific intensities using in-house server ‘tameNMR’ built within the galaxy toolkit (https://github.com/PGB-LIV/tameNMR). R Studio version 4.4.3 (www.r-project.org) and MetaboAnalyst 5.0 (www.metaboanalyst.ca, Canada) were used for statistical analysis. Multivariate analysis was performed, including principal component analysis (PCA) and partial least square discriminant analysis (PLS-DA). For supervised PLSDA, cross-validation was performed to determine model robustness on a training set of 70% of the data with area under the receiver operating curve (ROC) presented for each group with respect to all other groups in the model. A threshold of >1.0 variable importance in projection (VIP) score was used to assess the discriminating metabolites. To elucidate the relevance of metabolites and metabolic pathways, MetaboAnalyst 5.0 was used for pathway enrichment analysis. Once a list of metabolites was uploaded, enrichment analysis was performed using the Small Molecule Pathway Database and then subjected to over-representation analysis (ORA) using the hypergeometric test to evaluate whether a particular metabolite set is represented more than expected by chance within the given compound list. Based on the VIP loadings of PLS-DA analysis, only metabolites with a VIP > 2.0 were analyzed for enrichment, and one-tailed *p* values were used after adjusting for multiple testing. Multivariate analysis was performed, including principal component analysis (PCA) and partial least squares discriminant analysis (PLS-DA). To elucidate the relevance of metabolites and metabolic pathways, MetaboAnalyst 5.0 was used for pathway enrichment analysis [22].

### 2.5. Western Blotting

Proteins (20–30 µg) were resolved on a 10% SDS-polyacrylamide gel, transferred onto a nitrocellulose membrane, and probed with primary antibodies (anti-ChoK [Abcam; ab88053, Abcam, Cambridge, UK], anti-vinculin [Abcam; ab129002], anti-tubulin [Cell Signaling; 2128S, Cell Signaling technology Inc. Danvers, MA, USA], anti-E2F5 [Santa Cruz; sc-1083, Santa Cruz Biotechnology Inc, Dallas, TX, USA], anti-E2F1 [Cell Signaling; 3742], anti- α3+β1 [Abcam; ab217145], anti-HIF-1α [Protein Tech, Manchester, UK; 20960-1-AP], and anti-HIF-2α [Bethyl Laboratories, Montgomery, TX, USA; A700-003]) at 4 °C for 12 h. Membranes were incubated with anti-rabbit (1:10,000) horseradish peroxidase-linked secondary antibody (Cell Signaling Technology) for 2 h at room temperature. Signals were developed using Amersham ECL Prime Western blotting Detection Reagent (GE Healthcare, Chicago, IL, USA), and images were taken using a G:BOX gel imaging system (Syngene, Cambridge, UK).

### 2.6. Flow Cytometry

Cells were washed with phosphate-buffered saline (PBS), trypsinized, and resuspended in PBS at 0.5–1 × 10^6^/500 µL. Cells were fixed by adding them dropwise to ice-cold 70% ethanol, gently vortexing, and incubating for 30 min at 4 °C. The samples were washed 2× in ice-cold PBS and centrifuged for 10 min at 300× *g*. One milliliter of 0.5% Tween20/PBS was added to the sample pellets and incubated at room temperature for 5 min. The samples were washed 2× with ice-cold PBS, centrifuged for 3 min at 300× *g*, and all supernatants were removed. Immediately prior to data acquisition, samples were re-suspended in 1 mL of 1 µg/mL Hoechst 33342 in PBS (Invitrogen, Waltham, MA, USA, 10 mg/mL stock). Cell cycle analysis was performed using a BD Bioscience FACSCanto II flow cytometry system (BD Bioscience, Wokingham, Berkshire, UK) with a 401 nm laser. Flow cytometry data were analyzed using NovoExpress 1.4.1 software. The population of cells analyzed was gated according to Supplementary Figure S1 to ensure that only live singlets were included in the analysis, and NovoExpress built-in cell cycle analysis software was used to quantify the proportion of cells in G0/G1, S, and G2/M phases.

### 2.7. 3D Spheroids

A total of 1 × 10^6^ F98 H2B RFP or U-87 MG H2B RFP cells were seeded in 1 mL filtered culture media in one well of a 5D spherical plate (Kugelmeiers Ag, Erlenbach, Switzerland) and incubated at 37 °C overnight for spheroid formation. Spheroids were imaged on a Zeiss Light-Sheet microscope (Carl Zeiss Microscopy Ltd, Cambridge, UK) and analyzed using the software Imaris v9.6 software (www.imaris.oxinst.com; Oxford Instruments, Abingdon, Oxforshire, UK).

#### 2.7.1. Spheroid Formation

A total of 10 μL of spheroid/mounting media was drawn up into a fluorinated ethylene propylene (FEP) tube (S 1815-04, BOLA, Grunsfeld, Germany) mounted in 50% Matrigel, 40% filtered media, 10% 25 mM HEPES. For JAS239-treated spheroids, a final concentration of 500 nM JAS239 was added to the mounting media before addition to spheroids. To mimic hypoxic conditions, dimethyloxalylglycine (DMOG) was added to a final concentration of 0.5 mM in mounting media. The FEP tube was inserted into a 1.5 mm glass capillary (Brand GmbH, Frankfurt, Germany, catalogue #701908) and placed into the light-sheet sample holder, pre-set to 37 °C.

#### 2.7.2. Image Acquisition and Analysis

Spheroids were imaged on a Zeiss Light-Sheet microscope using a 561 nm laser for excitation and a 10× illumination objective. Emitted light was collected through a 576–615 nm filter using a 20× W Plan-Apochromat objective. A Z-stack of 7 µm steps was acquired every 3 min for 320 cycles. Light-Sheet Z.1 Zen software was used using dual-side fusion with a pivot scan. Images were detected using pco.edge scientific complementary metal–oxide–semiconductor (sCMOS) camera. Cell tracking was performed using Imaris v9.6 software (www.imaris.oxinst.com; Oxford Instruments, UK). A brief description of the image analysis is shown in Supplementary Figure S2. To discount any drift of the spheroids on tracking parameters, a reference frame was applied to each frame (Supplementary Figure S2A). Next, alignment of ‘spots’ (Supplementary Figure S2B) to each cell nuclei within the spheroid was applied, and the quality of spots was filtered using the following parameters: average size: 10 µM, max gap of 2 at 10 µM. These parameters were then applied to the whole data set, and ‘dragon tail’ tracks are shown (Supplementary Figure S2C) with the color scale denoting track duration. Data were filtered for all tracks greater than 2 h long, and various parameters were plotted, including average track speed, straightness, length, and mean squared displacement (MSD).

## 3. Results

### 3.1. JAS239 Efficacy Was Cell Line Dependant in Response to Hypoxic Conditioning

Since hypoxia influences drug effectiveness in various cancer models [23,24,25], we first assessed the effects of JAS239 on GBM cell metabolic activity under hypoxia exposure for 96 h (72 h of hypoxic preconditioning followed by 24 h JAS239 treatment under hypoxic conditions). No significant difference in IC50 was found between normoxic (21% O_2_) and hypoxic (1% O_2_) conditions across all four GBM cell lines after 24 h of JAS239 treatment (Supplementary Figure S3). Similar results were observed in cells preconditioned for 72 h but then reoxygenated for 24 h during JAS239 treatment (red lines). This experiment was designed to check if there was a direct chemical effect of the lack of oxygen on drug efficacy, as has been reported for other drugs, such as phleomycin [26]. JAS239 remained active and effective under all oxygenation conditions tested. JAS239 was also shown to increase *CHK* gene expression and ChoK protein expression in F98 and 9L normoxic cells (Supplementary Figure S4A–C). Conversely, *CHK* gene expression increased in U-87 MG and U-251 MG normoxic and hypoxic cells, whereas ChoK protein expression decreased with JAS239 treatment (Supplementary Figure S4A,D,E). Hypoxic exposure was monitored by measuring the HIF-1α protein levels. HIF-1α was indeed stabilized within 8 h of hypoxic exposure, and levels were maintained for 72 h in F98 and U-87 MG cells and to a lesser degree in 9L cells, indicating that the cells respond to hypoxia (Supplementary Figure S5A,B). Surprisingly, U-251 MG cells showed HIF-1α stabilization under normoxic and hypoxic conditions (Supplementary Figure S5B). HIF-2α protein levels also increased in the four cell lines under hypoxia (Supplementary Figure S5C,D). In both rat cell lines (F98 and 9L), there was detectable HIF-2α protein under normoxic conditions. Interestingly, treatment with JAS239 reduced HIF-1α and HIF-2α levels in all the cell lines after 72 h of hypoxia.

### 3.2. Metabolite Enrichment in JAS239 Normoxic and Hypoxic Cells

**Figure 1:**
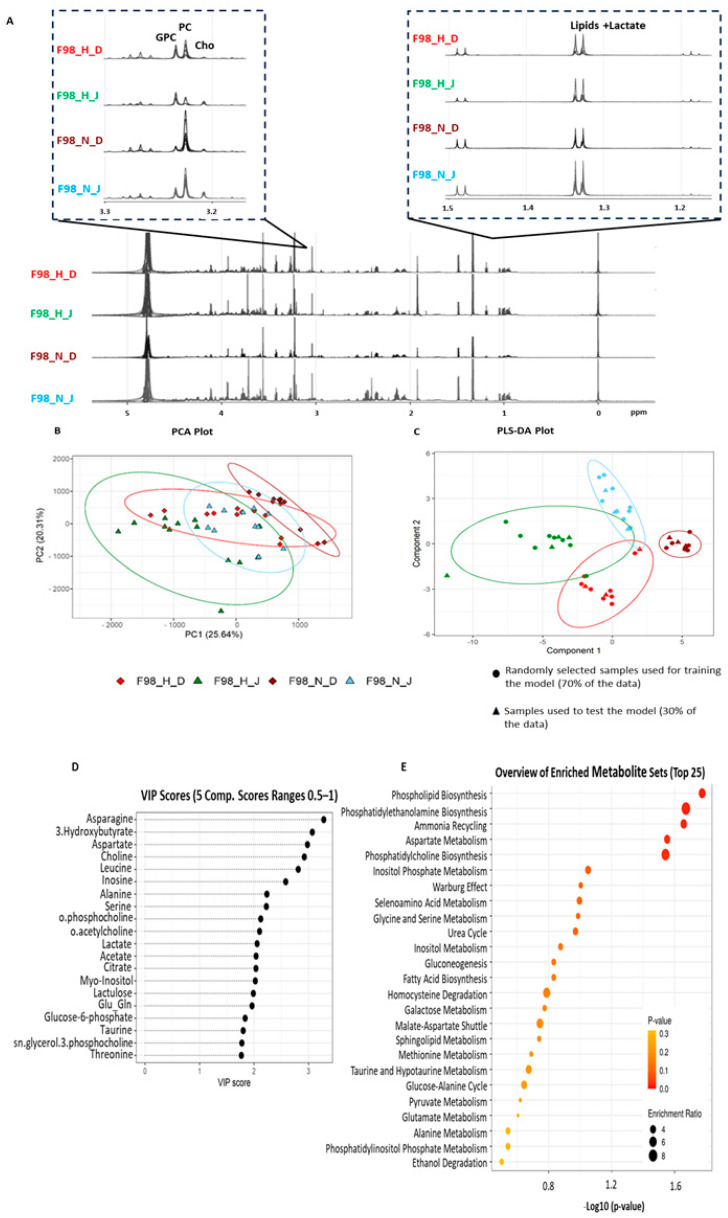
Representative figures for the metabolomic analysis pipeline. (**A**) Example F98 ^1^H 1D NMR spectra acquired at 700 MHz and referenced to the internal standard (TSP). (**B**) PCA analysis. (**C**) PLS-DA analysis (component 5 ROC scores for each group vs all other groups: H_D (red) = 1; H_J (green) = 1; N_D (purple) = 0.91 and N_J (blue) = 1) and (**D**) most influential metabolites. Metabolites with VIP > 2 (**D**) informed the metabolite set enrichment analysis of the top 20 metabolites using the hypergeometric test (**E**). D, DMSO; H, hypoxia; J; JAS239; N, normoxia; PCA, principal component analysis; PLS-DA, partial least square discriminant analysis; ROC, receiver operating characteristic; VIP, variable importance in projection coefficients.

We further established the intracellular metabolite modifications induced by JAS239 treatment. We focused on GBM metabolism using NMR-based metabolomic analysis because JAS239 targets the choline pathway by competitively binding to the active site of ChoK, preventing the phosphorylation of choline during phosphatidylcholine biosynthesis [16,18]. We hypothesized that phosphocholine levels would be reduced in JAS239-treated cells. The effect of prolonged hypoxia on phosphocholine levels is unknown. After 6 h of JAS239 treatment, a clear reduction in phosphocholine and an increase in glycerophosphocholine were detected under both normoxic and hypoxic conditions, as shown in the representative spectra of F98 cells (Figure 1A). Data transformation using PCA exhibited variance due to oxygenation as opposed to JAS239 treatment for all but U-251 MG cell lines and the loadings of metabolites that were most influential in the first and second principal components were identified and shortlisted (representative F98 PCA loadings are shown in Figure 1B). PCA of the F98 (Figure 1B) and 9L (Supplementary Figure S6A–D) cells showed that principal components 1 and 2 overlapped.

**Figure 2:**
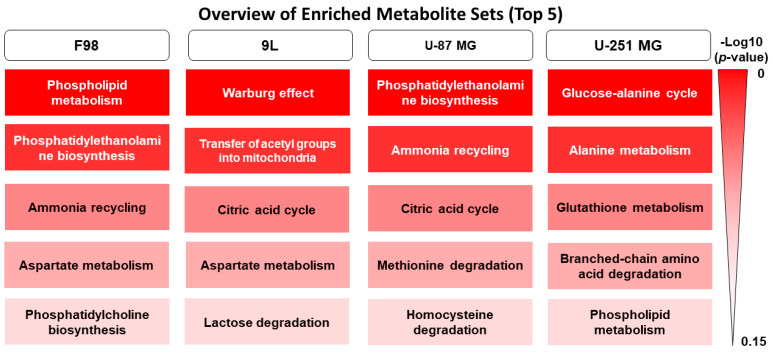
Top 5 enriched metabolic pathways. Metabolites with VIP > 2 informed the metabolite set enrichment analysis of the top 20 metabolites.

Phosphocholine, formate, and glycerophosphocholine were the most influential in PCA loadings of F98 cells, whereas acetate, phosphocholine, and creatine ranked the most influential in PCA loadings of 9L cells; however, this was most likely due to hypoxia alone rather than JAS239 treatment. Similarly, PCA analysis of U-87 MG cells (Supplementary Figure S7A–D) demonstrated separation between principal components 1 and 2 due to oxygenation, with glycerophosphocholine, formate, and choline ranking being the most influential, whereas the U-251 MG PCA analysis of principal components 1 and 2 completely overlapped (Supplementary Figure S8A–D). The shortlisted metabolites were then used to perform PLS-DA analysis between the treatment and hypoxic conditions (representative F98 Figure 1C). The PLS-DA models were optimally fit with four/five components and yielded well-defined separation between JAS239 and DMSO as well as hypoxic and normoxic conditions with component 5 ROC scores ranging for each group between 0.91–1 (representative F98 Figure 1C). F98 and 9L cells clearly discriminated between hypoxia/normoxia and DMSO/JAS239 treatment in both cell lines. As hypothesized, phosphocholine, glycerophosphocholine, and choline were listed among the VIP loading plots for F98 cells (Figure 1D) and phosphocholine and choline for 9L cells (Supplementary Figures S6B), but with differing influence, indicating that this pathway is influential in the separation of treatment conditions. Although the PLS-DA plot of components 1 and 2 of the 5-component models for both U-87 MG and U-251 MG datasets (Supplementary Figures S7B and S8B) did not show visible discrimination between groups, discrimination was visible in higher components of the model (U-87 MG component 5 ROCs: 0.74–1 and U-251 MG component 5 ROCs: 0.97–1). From the PLS-DA analysis, a shortlist of metabolites with a VIP score >1 was used to perform metabolite enrichment analysis (representative F98 Figure 1E) and the top five most influential metabolomic pathways altered in response to JAS239 and hypoxia are shown in Figure 2 for all cell lines. Enrichment analysis of F98 cells highlighted influential metabolites associated mostly with lipid metabolism pathways, particularly phospholipid metabolism. Conversely, enrichment analysis of metabolites most influential in 9L cells ranked the ‘Warburg effect’ followed by mitochondrial metabolism or the tricarboxylic acid (TCA) cycle. Pathway enrichment of U-87 MG data ranked phosphatidylethanolamine metabolism as the most influential, clearly indicating cell-line specificity in response to JAS239 treatment.

### 3.3. JAS239 Inhibits Cell Proliferation and Blocks Cell Cycle Progression, Regardless of Oxygenation, Except in the 9L Rat GBM Cell Line

**Figure 3:**
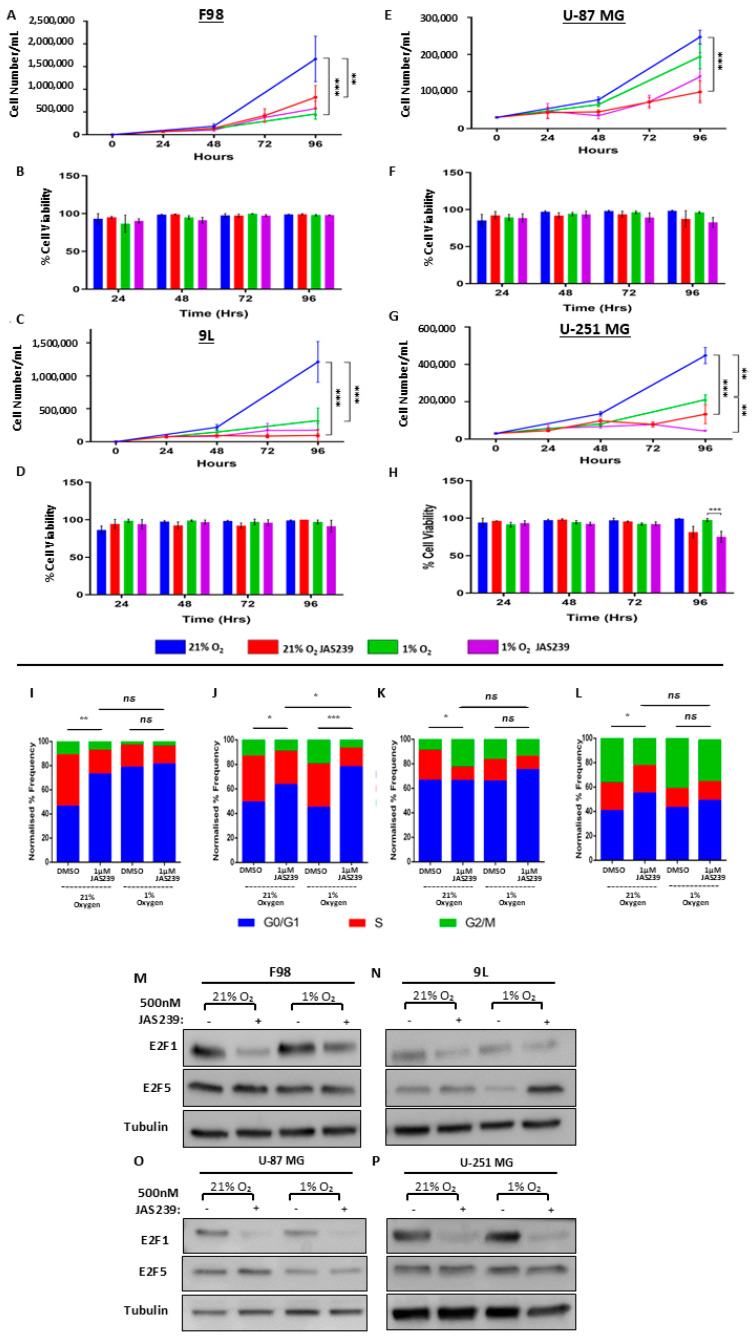
F98 (**A**,**B**), 9L (**C**,**D**), U-87 MG (**E**,**F**), and U-251 MG (**G**,**H**) cells were seeded and incubated in either 21% or 1% O_2_ and treated with either 500 nM JAS239 or 0.1% DMSO control. Cell counts and viability were collected for each respective time point (n = 3, +/− SEM). Multiple *t*-tests were performed on GraphPad Prism 9. Cell cycle distribution was acquired on a BD FACSCanto II system using a 405 laser and 440/50 nm emission filter. Analysis was performed using Novo Express 1.4.1 software, and cell cycle distributions were quantified. The histograms show the mean values of three experiments for F98 (**I**), 9L (**J**), U87 (**K**), and U251 (**L**) cells. 2-way ANOVA of multiple comparisons was performed on GraphPad Prism. * *p* < 0.05, ** *p* < 0.01, *** *p* < 0.001. Protein expression of E2F1 and E2F5 are shown for F98 (**M**), 9L (**N**), U-87 MG (**O**), and U-251 MG (**P**). ns, non-significant.

We further aimed to probe the effect of JAS239 on cell proliferation under normoxic or hypoxic exposure. Based on the dose–response curves (Supplementary Figure S3), we predicted that JAS239 exhibits similar effects on proliferation under normoxic and hypoxic conditions. We observed that F98 and 9L cells displayed a significant reduction in proliferation at 96 h in the presence of JAS239 under normoxic conditions (Figure 3A,C) without affecting cellular viability (Figure 3B,D). Hypoxia also affects rat GBM cell proliferation in a manner comparable to that of JAS239. There was no additive effect of the combination of the two conditions on F98, yet 9L cell proliferation was further reduced in the presence of both hypoxia and JAS239. In contrast, U-87 MG cell proliferation was unaffected by hypoxic conditions (Figure 3E). U-251 MG cells showed the strongest reduction in proliferation when treated with JAS239 under normoxic conditions (Figure 3G). JAS239 appeared to be slightly less effective in inhibiting the proliferation of hypoxic U-251 MG cells. In this case, the reduction in U-251 MG cell number over time was most likely due to induced cell death after 96 h of drug treatment, given the significant reduction in cell viability (Figure 3H). U-87 MG cell viability (Figure 3F) was also reduced with JAS239 treatment for 96 h; however, this was not significant.

To further validate the proliferation data and to determine if the JAS239-induced reduction in proliferation was due to cell cycle arrest, we investigated the cell cycle distribution of GBM cells in response to JAS239 treatment. Cell cycle distribution in viable single-cell populations was determined using flow cytometry. We observed a significant increase in cells in the G0/G1 phase in F98 (*p* < 0.01, 72.3 ± 11.5%), 9L (*p* < 0.05, 63.4 ± 11.3%), and U-251 MG (*p* < 0.05, 22.6 ± 5.9%) cells treated with JAS239 (Figure 3I,J,L). Concomitantly, we also observed a significant reduction of cells in the S phase for F98 (*p* < 0.01, 19.6 ± 8.7%) and U-87 MG cells (*p* < 0.05, 10.8 ± 2.6%; Figure 3I,K) and a reduction of cells in the G2/M phase in U-251 MG cells (*p* < 0.05, 22.6 ± 5.9%; Figure 3L). Similar to the proliferation data, the effects of JAS29 were comparable under normoxic and hypoxic conditions (Figure 3I,K,L), in agreement with the MTT data (Supplementary Figure S3). The exception was 9L cells, where the combination of JAS239 and hypoxia had an enhancing effect on cell cycle arrest in G0/G1, with a significant increase in cells in G0/G1 between normoxic and hypoxic cells treated with JAS239 (63.4 ± 11.2% versus 44.8, ±6.7%, respectively, *p* < 0.001). To elucidate the molecular mechanisms underlying the proliferation effects, we measured the protein expression levels of the E2F transcription factors, E2F-1 and 5. E2F-1 is a promoter of the G0/G1 to S phase transition, and E2F-5 is considered a repressor of cell cycle progression [27]. We observed a decrease in E2F-1 protein levels upon JAS239 treatment in all cell lines, regardless of oxygen levels (Figure 3M–P), in line with the cell proliferation and cell cycle analysis above (Figure 3I–L). Notably, the protein levels of the cell cycle repressor E2F-5 were comparable regardless of the conditions (JAS239 treatment and oxygen levels), with the notable exception of hypoxic JAS239-treated 9L cells, where a drastic increase in protein expression was observed. This observation is consistent with the cell cycle analysis results (Figure 3J), where the most significant effect in these cells was observed with hypoxia and JAS239 combination.

### 3.4. Inhibition of Cell Invasion/Motility by JAS239 Is Cancelled by Hypoxia

While the effects of JAS239 on cell proliferation have been previously explored in different tumor types [16,18], we wanted to determine whether JAS239 could also affect cell motility and invasive properties of GBM. We used a physiological 3D spheroid model to assess invasion, as it is difficult to assess this phenotype in cells grown as monolayers. The effects of JAS239 on cell invasion and motility were determined in two GBM cell lines: F98 (rat) and U87-MG (human). We first assessed drug diffusion and distribution throughout spheroids using the fluorescent properties of the drug. As the incubation system on the light sheet microscope did not allow us to decrease oxygen levels, we used the hypoxia-mimetic drug DMOG as a proxy for hypoxic conditions.

**Figure 4:**
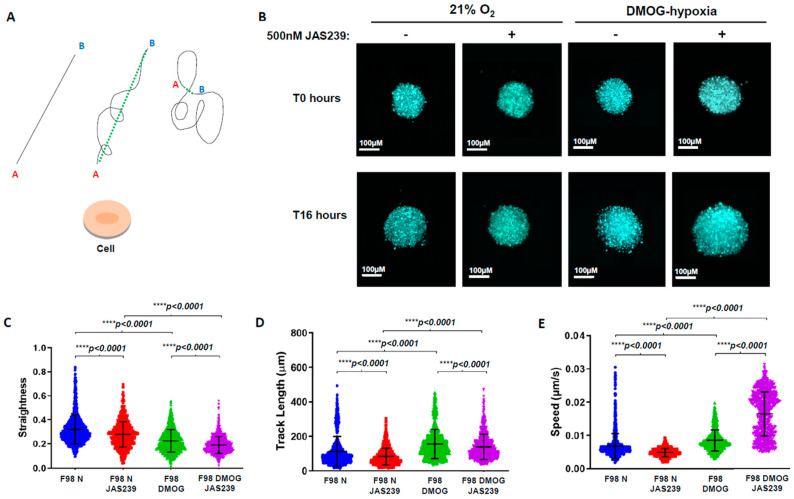
Schematic representation of the different types of cell movements and how each feature can be measured using Imaris software (**A**). The black lines show the potential directions and tracks that a cell could take from position A to position B. Track length is a measure of the black lines in these examples. The dashed green line represents the displacement of the cell, irrespective of the ‘distance’ the cell has moved. Track straightness and track speed can be determined using the track length. Spheroids were formed using 1.5 × 10^6^ F98 H2B RFP labeled cells and were incubated for 24 h at 37 °C/5% CO_2_. To mimic hypoxic conditioning, the prolyl hydroxylase inhibitor, DMOG (0.5 mM) was added to the matrigel:media:hepes matrix during the spheroid mounting. For the JAS239 treatment, a final concentration of 500 nM was applied to the mounting media. Once mounted into FEP tubing, spheroids were left for 1 h to immobilize before loading onto the Z.1 Light-Sheet microscope. A further 4 h of setting time in the microscope chamber was allowed before the experiment started. Z-stacks of 6.5 µM in thickness every 3 min for 16 h were acquired. (**B**) Representative spheroid pictures at time zero (T0) and 16 h (T16) in both 21% and 1% O_2_, +/− 500 nM JAS329. Using Imaris v6.9, tracks were filtered for a minimum of 2 h in length (n = 3) and track straightness (**C**), track length (**D**) and speed (**E**) were assessed. A *t*-test with Welch’s correction was performed using GraphPad Prism. ns, non-significant.

Similar to cells cultured in a 2D monolayer, JAS239 was localized in the cell cytoplasm (Supplementary Figure S9A–C); however, its diffusion throughout all cells within the 3D spheroid took approximately 77 min longer (80 min in the spheroid versus 3 min in cell monolayers, Supplementary Figure S9D,E). JAS239 remained inside cells for at least 48 h. A cell track analysis was then used to measure the position of the cell nuclei within the spheroid, and we determined typical motility features, including speed, track length, straightness, and displacement (dashed green line; Figure 4A) over 16 h time-lapse experiments.

**Figure 5:**
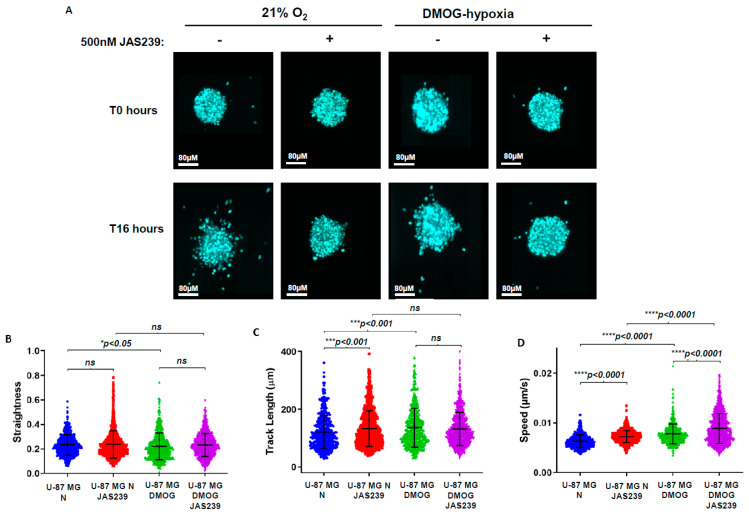
Spheroids were formed using 1.5 × 10^6^ U-87 MG H2B-RFP labeled cells and were incubated for 24 h at 37 °C/5% CO_2_. To achieve hypoxic conditioning, the prolyl hydroxylase inhibitor, DMOG (0.5 mM), was added to Matrigel:media:hepes matrix upon spheroid mounting. For the JAS239 treatment, a final concentration of 500 nM was applied to the mounting media. Once mounted into FEP tubing, spheroids could be set for 1 h before loading onto the Light-Sheet microscope and a further 4 h on the microscope before the experiment started. Z-stacks of 6.5 µM in thickness every 3 min for 16 h were acquired. Screenshots (**A**) of representative spheroid pictures at time zero (T0) and 16 h (T16) in both 21% and 1% O_2_, +/− 500 nM JAS329. Using Imaris v6.9, tracks were filtered for a minimum of 2 h in length (n = 3) and track straightness (**B**), track length (**C**) and speed (**D**) were assessed. A *t*-test with Welch’s correction was performed using Graphpad Prism. ns, non-significant.

The effect of JAS239 on 3D cell motility was visually striking in the time-lapse videos, as shown in the representative snapshots of F98 (Figure 4B) and U-87 MG (Figure 5A) spheroids. In comparison to untreated controls, fewer cells invaded the surrounding matrix after 16 h, and the spheroids appeared much more compact, indicating that JAS239 reduced the invasive properties.

**Figure 6:**
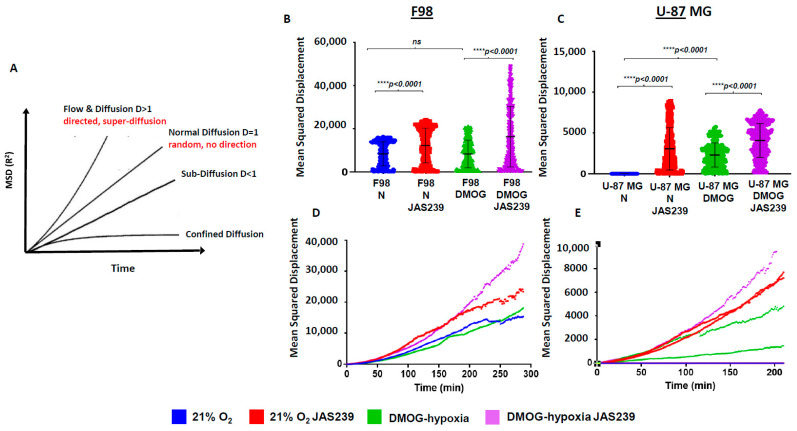
Schematic representation of the MSD (R^2^) versus time for diffusion and flow, normal diffusion, sub-diffusion, and confined diffusion (**A**). D, self-diffusion coefficient; figure adapted from Saxton and Jacobson, 1997. Mean Squared Displacement (MSD; R^2^) analysis of F98 (**B**,**D**) & U-87 MG (**C**,**E**) H2B RFP labeled spheroids were assessed using Imaris v6.9 for tracks at least 2 h in length. Dot plots of combined MSD are shown for F98 (**A**) and U-87 MG spheroids in control and DMOG treated (0.5 mM) with 500 nM JAS239. MSD trajectories for F98 (**D**) and U-87 MG (**E**) spheroids are also shown +/− SEM, n = 3. MSD, mean squared displacement.

To quantify this effect and assess cell displacement, we used the mean squared displacement (MSD), which is a measure of the average deviation of a particle from a reference point over time [28]. The type of diffusion can be determined from the pattern of the MSD trajectory and the value of the self-diffusion coefficient (D), as illustrated in Figure 6A. The MSD values (Figure 6B,C) and MSD plots for F98 and U-87 MG spheroids showed an increased slope with the addition of JAS239 in normoxic and DMOG conditions (Figure 6D,E), indicating that JAS239 induced a more directed ‘super-diffusion’ motion in the cells. However, we postulate this displacement must be within the spheroid mass as opposed to the periphery of the spheroid, given that fewer cells invaded the surrounding matrix.

To further evaluate the overall cell movement in the F98 and U-87 MG spheroids, we measured several features, including track speed, straightness, and length, which, combined, are more informative of the cells’ behavior. JAS239 significantly reduced track length in F98 spheroids compared to untreated controls (113.2 ± 86 µm versus 84.0 ± 48.0 µm; *p* < 0.0001, Figure 4D), but in contrast, induced an increase in track length in U-87 MG spheroids (120.7 ± 57.7 µm versus 133.0 ± 61.6 µm; *p* < 0.001 Figure 5C). Track straightness provides better information on motility/invasiveness through the division of displacement over the track length. A track straightness value closer to zero implies more disorganized movement, whereas a value closer to one implies a straighter path. F98 cells showed a significant reduction in track straightness with JAS239 treatment (control: 0.32 ± 0.12; JAS239:0.28 ± 0.11; *p* < 0.0001, Figure 4C) whereas no changes in straightness were observed for U-87 MG (Figure 5B). JAS239 significantly reduced the average speed of cells in F98 spheroids (control: 0.007 ± 0.004 µm/s, JAS239:0.005 ± 0.001 µm/s; *p* < 0.0001, Figure 4E), however the opposite effect was observed in U-87 MG JAS239 treated spheroids (control: 0.006 ± 0.001 µm/s; JAS239:0.007± 0.001 µm/s, *p* < 0.0001, Figure 5D). This suggests that JAS239 affects cell motility in a cell-line-dependent manner. Next, we determined the impact of hypoxia on JAS239 treatment on these cell motility parameters. Compared to controls, the addition of DMOG significantly reduced F98 and U87 track straightness and increased track length and speed (Figure 4C–E *p* < 0.0001 and Figure 5B–D *p* < 0.05, respectively). The addition of JAS239 to DMOG treatment significantly reduced F98 cell track straightness and length and, surprisingly, increased cell speed (Figure 4C–E; *p* < 0.0001). Track straightness and speed in DMOG and JAS239 treated U-87 MG cells were comparable to DMOG only (Figure 5B,D); however, similar to F98 spheroids, there was a significant increase in track speed in the presence of both DMOG and JAS239 (Figure 5D).

Collectively, these data suggest that JAS239 affects key GBM cell motility parameters and reduces the invasive potential of GBM cells into the surrounding matrix, and its effects vary between the control and DMOG conditions. In F98 spheroids, DMOG and JAS239 treatment induced faster, more super-diffusive motion than JAS239-only spheroids, but both conditions induced disorganized cell movements, with significantly shorter track lengths. Similarly, in U-87 MG spheroids, DMOG and JAS239 treatment induced faster and more super-diffusive motion than spheroids treated with JAS239 alone.

To better explain the invasion parameters, we assessed the protein expression of integrin α3β1 as a marker of cell mobility since α3β1 mediates the migration of neuronal cells [29]. We observed reduced α3β1 levels in F98 (Supplementary Figure S10A), 9L (Supplementary Figure S10B), and U-251 MG (Supplementary Figure S10D) cells treated with JAS239, consistent with the decreased invasion observed in the spheroids. However, U-87 MG cells showed no change in α3β1 expression (Supplementary Figure S10C), suggesting that other cell migration signaling molecules may be involved in the reduction in cell invasion previously observed upon JAS239 treatment (Figure 5).

## 4. Discussion

In this study, we explored the effects of the hypoxic microenvironment on GBM cell response to ChoK inhibition. In addition to the classical effects expected from drugs affecting cellular metabolism (cell proliferation, survival, and cell cycle progression), we also assessed cell mobility and invasion. Interestingly, the ChoK inhibitor, JAS239, which is typically assessed for its antiproliferative effects in tumor cells, also demonstrated clear inhibitory effects on cell invasion. This observation, along with the fact that JAS239 potency was not damped by hypoxic conditions to which tumor cells are exposed, makes this drug an interesting candidate for GBM treatment as it has the potential to not only reduce tumor growth but also its invasion, which remains one of the major clinical challenges in the treatment of GBM. Additionally, our work on different GBM cell lines shows an important cell line-to-cell line variability, which must be considered for future potential use in the clinic.

### 4.1. JAS239

The effects of JAS239 have not been explored in hypoxic tumor cells. Under normoxic conditions, JAS239 induced a significant reduction in proliferation via cell cycle arrest in all cell lines, which was expected given its effects in reducing phosphatidylcholine synthesis used for cell membrane synthesis. Under hypoxic conditions, we observed no synergistic effect of JAS239 on cell proliferation or cell cycle arrest. Yet, we found JAS239 to be equally efficacious under hypoxic conditions with respect to reducing cell invasion which, given the high mortality of GBM being largely attributed to its highly invasive nature, makes it a promising treatment strategy for GBM patients.

The effects of other choline kinase inhibitors on cancer cell migration and invasion have been previously reported. EB-3D-mediated ChoK inhibition significantly reduced the migratory and invasive properties of the highly aggressive MDA-MB-231 breast cancer cell line [30]. Although these cell invasion assays were carried out in 2D transwell plate inserts, these data are in line with what we observed in highly aggressive GBM lines using JAS239 in 3D spheroid models. Interestingly, Mariotto et al. also demonstrated reduced in vivo lung metastases with inhibition of ChoK, providing further evidence of the potential of ChoK inhibitors as effective treatments for aggressive and highly invasive cancers [30]. However, these drugs will probably only reach their full promise when used in combination with other therapies. For example, the inhibition of ChoK has been shown to increase the sensitivity of aggressive endothelial ovarian cancer cells to commonly used chemotherapeutics, including paclitaxel and doxorubicin, in breast cancer cells [31,32].

The efficacy of JAS239 treatment has also been demonstrated in in vivo rodent models of breast cancer and GBM [16,33]. Initial studies by Arlauckas et al. reported JAS239 efficacy with a significant reduction in total choline levels and tumor growth in a breast cancer xenograft model [16]. Additionally, in previous in vivo experiments, we used ^1^H NMR spectroscopy (^1^H MRS) to assess JAS239 efficacy in three GBM xenograft rodent models (GL261, F98, and 9L) and demonstrated a reduction in total choline levels in all three models in response to JAS239, with the 9L intracranial tumors exhibiting the largest reduction [33]. Interestingly, the opposite trend was observed for tumor volume; when compared to baseline, the largest tumor growth arrest was found in GL261 and F98 tumors, followed by 9L tumors [33]. These in vivo data support the observations made in the current study with respect to JAS239-induced reductions in proliferation via cell cycle arrest, with no effect on cell viability. The in vitro data obtained in this study also explains why only tumor growth arrest was observed under in vivo conditions, as opposed to a reduction in tumor volume [21]. Moreover, the largest reduction in choline levels occurred within the tumor region, which coincided with reductions in the mitotic index for all three GBM models, suggesting that JAS239 selectively inhibits proliferating cells within the tumor, where ChoK is overexpressed.

### 4.2. Hypoxia and GBM

Hypoxia is an important hallmark of GBM and is attributed to its highly aggressive and invasive phenotype. Adaptation to hypoxia is mediated via HIF-1/2α and typically involves the reduction of high-energy-consuming pathways, such as cell division [13,34]. How cells respond to hypoxia can be dependent on the severity and duration of hypoxia conditioning; however, most cells respond via arresting at the G1 or early S phase of the cell cycle [35]. The exact mechanism is disputed across the literature, as some studies suggest that G1/S arrest is due to a hypoxia-induced increase in p27 levels [36]. In contrast, a study investigating the effects of hypoxia on the cell cycle response across multiple cell lines reported only one cell line exhibiting a hypoxia-induced increase in p27 mRNA and two at the protein level. HeLa cells showed a reduction in p27 in response to hypoxia but were arrested in the G1/S phase [35]. However, the authors did note the cell type-dependent response to hypoxia. Our data are very similar to previous observations by Richards et al., where a panel of human GBM cell lines (D566, U-87 MG, and U-251 MG) was assessed at varying oxygen concentrations (0.1% O_2_ and 1% O_2_) for 24–72 h, with no change in cell cycle distribution even at 0.1% O_2_. No changes in the expression levels of p21, p27, and the G1/S transition transcription factor E2F1 were reported in that study [34]. Our data confirmed the cell type-dependent response to hypoxia. Indeed, out of the four cell lines tested, only F98 cells demonstrated significant G0/G1 cell cycle arrest after 96 h of hypoxia conditioning; the other cell lines appeared to have more cells in the G2/M phase, yet this was not significant. Overall, rat GBM cells appeared more sensitive to prolonged hypoxia compared to both human GBM lines; however, U-87 MG cells were notably the least sensitive of all cells. Since cell adaptation to hypoxia is mediated by HIF-1/2α, we assessed the protein stability of HIF-1/2α over time. Of the cell lines studied, U-87 MG cells showed the highest HIF-1α stabilization between 24 and 72 h, whereas U-251 MG cells showed consistent HIF-1α stabilization even under normoxic conditions. The differences in HIF-1α protein expression over time may explain the differences in the cell-specific sensitivity to hypoxia. Another potential mechanism by which hypoxia induces pro-tumorigenic effects is through the enhancement of tumor invasion [37,38]. In a study assessing U-87 MG and U-251 MG migration and invasion, cells conditioned for 72 h under hypoxia (1% O_2_) demonstrated drastic differences in migration potential. The increase in the migration potential of U-251 MG cells was limited compared to that of U-87 MG cells, where migration doubled in a wound healing assay [39]. In our U-87 MG spheroid invasion experiments, we observed a significant increase in track straightness, length, and speed between normoxia and DMOG-hypoxia. Additionally, we observed an increase in the MSD with DMOG treatment, indicative of super-diffusion motion. Joseph et al. correlated this hypoxia-induced increase in the migratory potential of cells with the mesenchymal transition mediated by HIF-1α and ZEB1 [39]. GBMs with a mesenchymal signature are suggested to be of particular importance as they are deemed to be highly aggressive compared to the other subtypes, proneural, neural, and classical, and are considered to be highly resistant to therapies [40,41]; U-87 MG cells are considered to be a model for the mesenchymal subtype of GBM.

### 4.3. JAS239 and Hypoxia

An unexpected but promising finding of this study was the JAS239-induced reduction in HIF-1/2α protein levels across all cell lines. Given the vast number of protumorigenic genes regulated by HIF transcription factors and their association with treatment resistance [42,43], this indirect effect of JAS239 adds to the potential of this inhibitor in hypoxic tumors. Several direct and indirect HIF inhibitors (panzem, tanespimycin, zolinza) have been tested in phase I/II clinical trials of multiple myeloma, ovarian cancer, prostate cancer, and lymphoma; however, these trials have not reported significant improvements in overall survival and modest progression-free survival compared to the current standard of care [44]. The potential of JAS239 as an inhibitor of HIF, in addition to its metabolic, anti-proliferative, and anti-invasive properties, warrants further investigation and could serve as a candidate treatment in combination with standard-of-care treatments in GBM to target multiple aspects of tumorigenesis.

## 5. Conclusions

While we previously demonstrated the anti-tumor activity of JA239 in vivo [33], we provide further understanding of its molecular mechanisms of action, including changes in intracellular metabolites and cellular effects on proliferation and invasion. We noticed an important cell line-to-cell line variability, likely representative of the clinical heterogeneity of these tumors, displaying a range of molecular subtypes and mutational burdens. Due to this heterogeneity, human GBMs have been categorized into defined molecular subgroups. Our findings on the ability of a single drug (JAS239) to obliterate at once at least three different hallmarks of cancer and one enabling characteristic [45]: unlimited proliferation, deregulating cellular metabolism, activating invasion, and activating HIF opens the way for new promising strategies that can act on multiple pro-tumorigenic features, increasing the chance of better eradicating tumor development in patients.

## Data Availability

All the raw data files presented in this study are available upon request from the corresponding author. The NMR data files from the study are available at https://www.ebi.ac.uk/metabolights/MTBLS6212/ID MTBLS6212.

## Supplementary Materials

The following supporting information can be downloaded at: https://www.mdpi.com/article/10.3390/metabo15020076/s1, Figure S1. Flow cytometry data was analysed using NovoExpress 1.4.1 software. The population of cells analysed were gated to ensure only live singlets were included in the analysis and NovoExpress built-in cell cycle analysis software was used to quantify the proportion of cells in G0/G1, S and G2/M phase. Figure S2. MTT of F98 (A) 9L (B) U-87 MG (C) U-251 MG (D) dose response curves upon JAS239 treatment. Half-log serial dilutions of JAS239, starting from 100µM were applied to F98, 9L, U-87 MG and U-251 MG cells that had either been preconditioned in 21% O_2_ or 1% O_2_ for 3 days. JAS239 was applied for 24 hours in either 21% O_2_ (blue) or cells that were previously incubated in 1% O_2_ for 3 days and then reoxygenated during JAS239 treatment (red) or cells preconditioned for 3 days and then treated in 1% O_2_ for an additional 24h (green). Cellular metabolic activity was measured by MTT. Data were normalized to DMSO control (n = 3). Figure S3. JAS239 significantly increased CHK gene expression (A) in all cell lines under normoxic conditioning (F98 *p* < 0.0001, 9L *p* < 0.001, U-87 MG *p* < 0.0001 and U-251 MG *p* < 0.05). Only U-87 MG (*p* < 0.01) and U-251 MG (p<0.05) cells had significant increase in gene expression under hypoxic conditioning. Protein expression of F98 (B) and 9L (C) showed increased ChoKα expression with JAS239 treatment in normoxic conditioning only. U-87 MG (D) protein expression decreased with JAS239, most notably in normoxic conditioning and U-251 MG cells (E) demonstrated a reduction in hypoxic conditioning only. Figure S4. HIF-1α and HIF-2α protein expression was assessed over 72 hours and shows an induction in HIF-1α in response to hypoxia in all cell lines apart from U251 cells, where HIF-1α expression was already present in 21% O_2_, and HIF-2α induction in all cell lines. 500 nM JAS239 was added for 24 hours at the 72-hour time point. HIF-1α levels were reduced in all cell lines in presence of JAS239 (n = 3). Figure S5. 9L metabolomic analysis pipeline. (A) PCA analysis, (B) PLS-DA analysis (component 4 ROC scores for each group vs. all other groups: H_D = 1; H_J = 0.96; N_D = 1 and N_J = 0.96) and (C) most influential metabolites. Metabolites with VIP > 2 (C) informed the metabolite set enrichment analysis of top 20 metabolites using the hypergeometric test (D). Figure S6. U-87 MG metabolomic analysis pipeline. (A) PCA analysis, (B) PLS-DA analysis (component 5 ROC scores for each group vs. all other groups: H_D = 0.74; H_J = 1; N_D = 0.97 and N_J = 1) and (C) most influential metabolites. Metabolites with VIP > 2 (C) informed the metabolite set enrichment analysis of top 20 metabolites using the hypergeometric test (D). Figure S7. U-251 MG metabolomic analysis pipeline (A) PCA analysis, (B) PLS-DA analysis (component 5 ROC scores for each group vs. all other groups: H_D = 0.97; H_J = 1; N_D = 1 and N_J = 1) and (C) most influential metabolites. Metabolites with VIP > 2 (C) informed the metabolite set enrichment analysis of top 20 metabolites using the hypergeometric test (D). Figure S8. U-87 MG Zs Green labelled cells (green) were imaged on an Andor Dragonfly every 30 s for 2 h in either a 2D (A–C) or 3D (D) model using 40x oil-based objective. 500 nM JAS239 (red) was applied after the first z-stack was acquired. Image analysis was done using Imaris, after background subtraction, fluorescent intensity over time was plotted; FOV = 3, n = 3 (E). Figure S9. Cells were exposed to 21% or 1% O_2_ for 96 hours before extraction. Protein expression of α3β1 for all cell lines are visible A–D. Figure S10. Imaris V9.6 was used to analyse cell straightness, speed, track length, and mean- squared displacement. To account for spheroid drift, a reference frame is added to all frames (A) before spots are added to the cell nuclei (B) which can be filtered based on size and gap distance between frames. Once these parameters have been applied to the whole data set, track length can be observed with ‘dragon tail’ tracks (C), the colour code inferred to the track duration.
