# Supplementary material for: Hypoxia Dependent Inhibition of Glioblastoma Cell Proliferation, Invasion, and Metabolism by the Choline-Kinase Inhibitor JAS239
Source: Metabolites. 2025 Jan 26;15(2):76. doi: 10.3390/metabo15020076 (PMC11857610; doi:10.3390/metabo15020076)
Supplement: Supplementary file 1 [file metabolites-15-00076-s001.zip › metabolites-3408062-supplementary.pdf]

Supplementary Figures

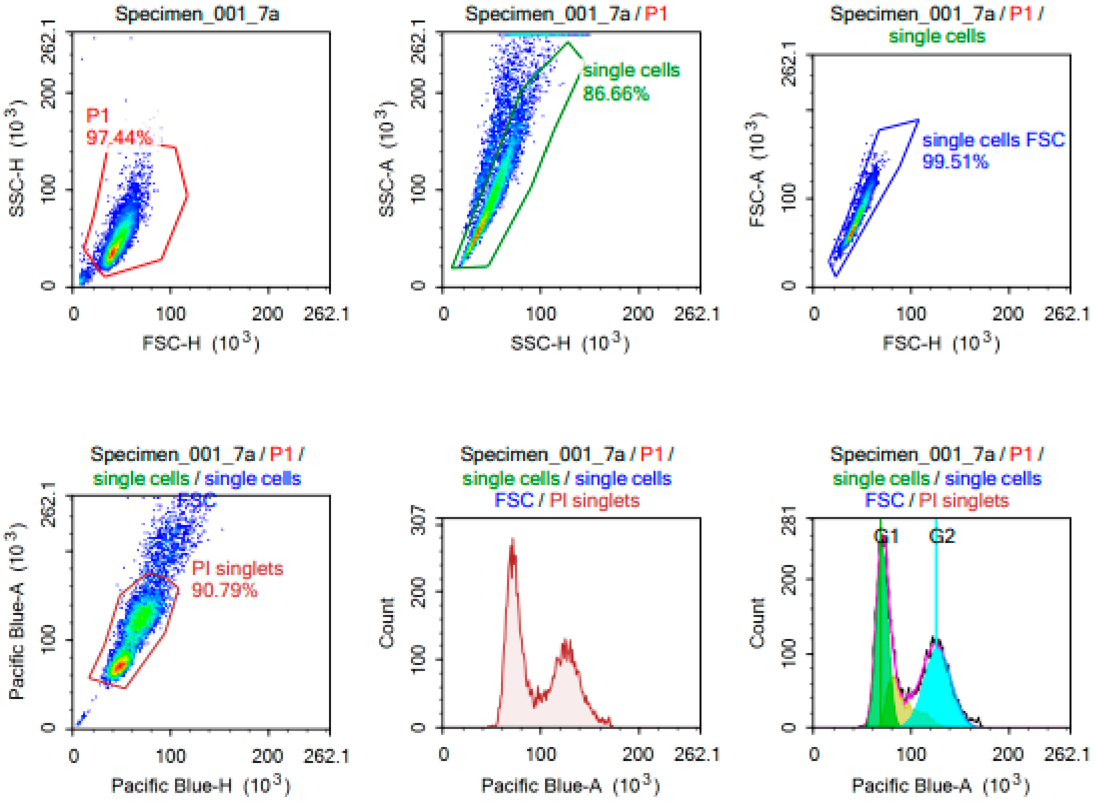

Figure S1. Flow cytometry data was analysed using NovoExpress 1.4.1 software. The population of cells analysed were gated to ensure only live singlets were included in the analysis and NovoExpress built-in cell cycle analysis software was used to quantify the proportion of cells in G0/G1, S and G2/M phase.

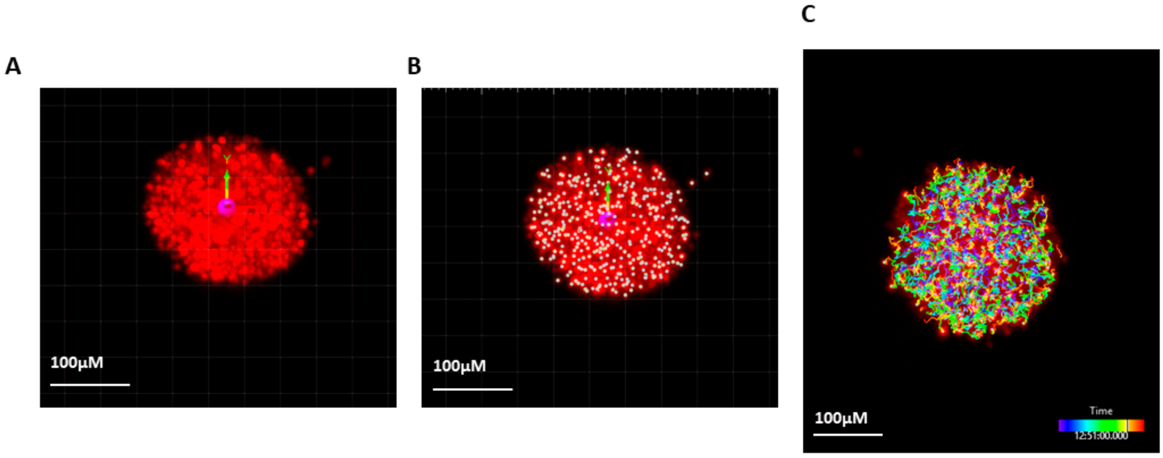

Figure S2. Imaris V9.6 was used to analyse cell straightness, speed, track length, and mean- squared displacement. To account for spheroid drift, a reference frame is added to all frames (A) before spots are added to the cell nuclei (B) which can be filtered based on size and gap distance between frames. Once these parameters have been applied to the whole data set, track length can be observed with ‘dragon tail’ tracks (C), the colour code inferred to the track duration.

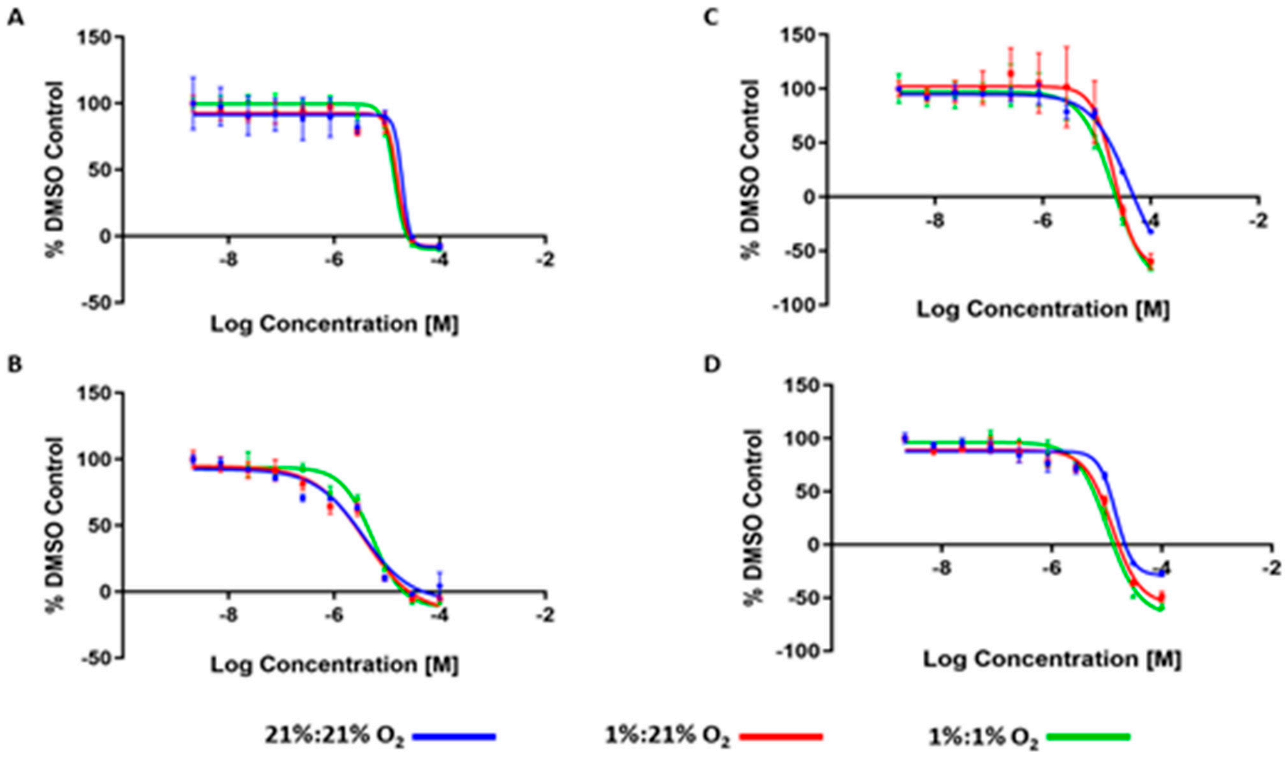

A
B
C
D
150
100
50
0
-50
-100
% DMSO Control
-8
-6
-4
-2
Log Concentration [M]
21%:21% O_2
1%:21% O_2
1%:1% O_2

Figure S3. MTT dose response curves upon JAS239 treatment of F98 (A) 9L (B) U-87 MG (C) U-251 MG (D). Half-log serial dilutions of JAS239, starting from 100µM were applied to F98, 9L, U-87 MG and U-251 MG cells that had either been preconditioned in 21% O_2 or 1% O_2 for 3 days. JAS239 was applied for 24 hours in either 21% O_2 (blue) or cells that were previously incubated in 1% O_2 for 3 days and then reoxygenated during JAS239 treatment (red) or cells preconditioned for 3 days and then treated in 1% O_2 for an additional 24h (green). Cellular metabolic activity was measured by MTT. Data were normalized to DMSO control; N=3.

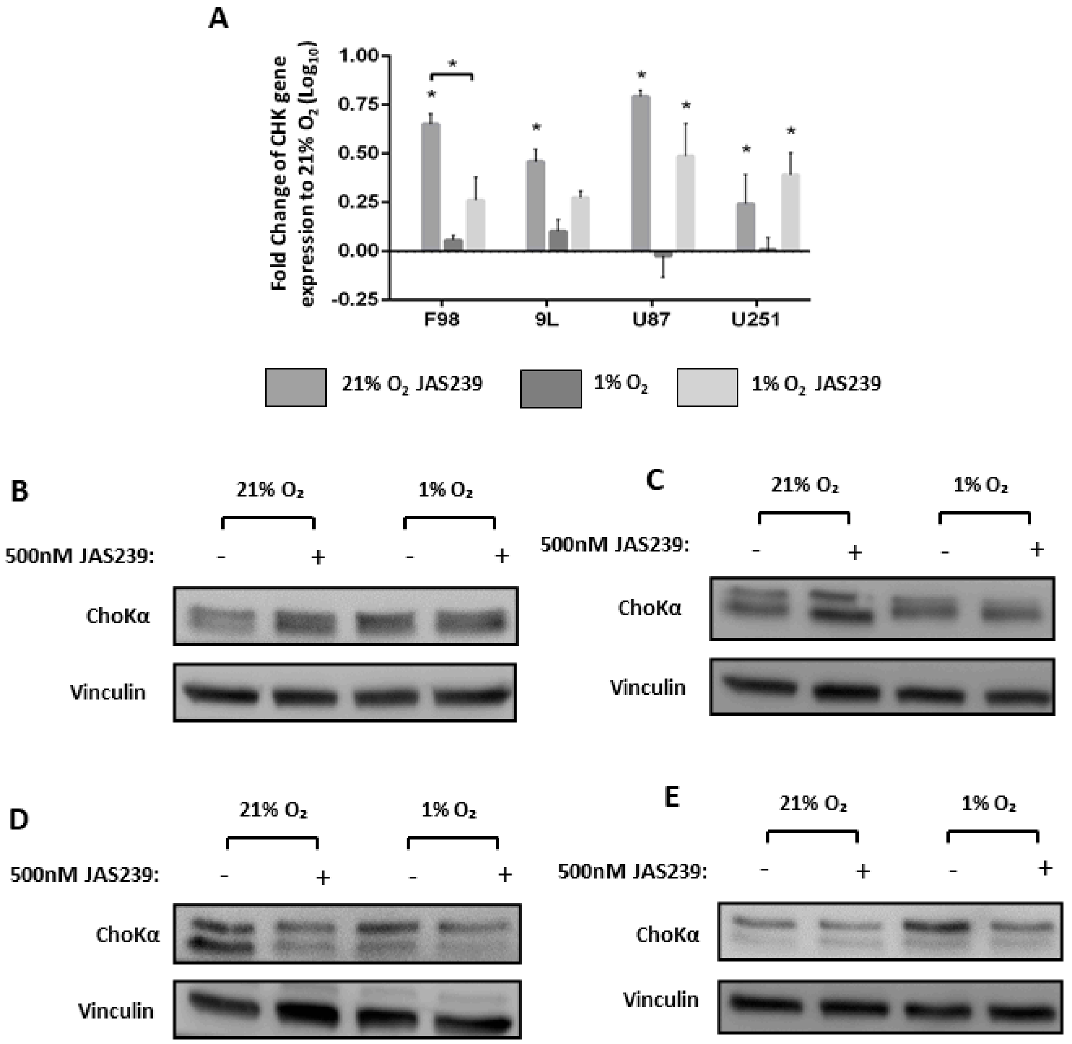

Figure S4. JAS239 significantly increased CHK gene expression (A) in all cell lines under normoxic conditioning (F98 $p<0.0001$, 9L $p<0.001$, U-87 MG $p<0.0001$ and U-251 MG $p<0.05$). Only U-87 MG ($p<0.01$) and U-251 MG ($p<0.05$) cells had significant increase in gene expression under hypoxic conditioning. Protein expression of F98 (B) and 9L (C) showed increased ChoK*α* expression with JAS239 treatment in normoxic conditioning only. U-87 MG (D) protein expression decreased with JAS239, most notably in normoxic conditioning and U-251 MG cells (E) demonstrated a reduction in hypoxic conditioning only.

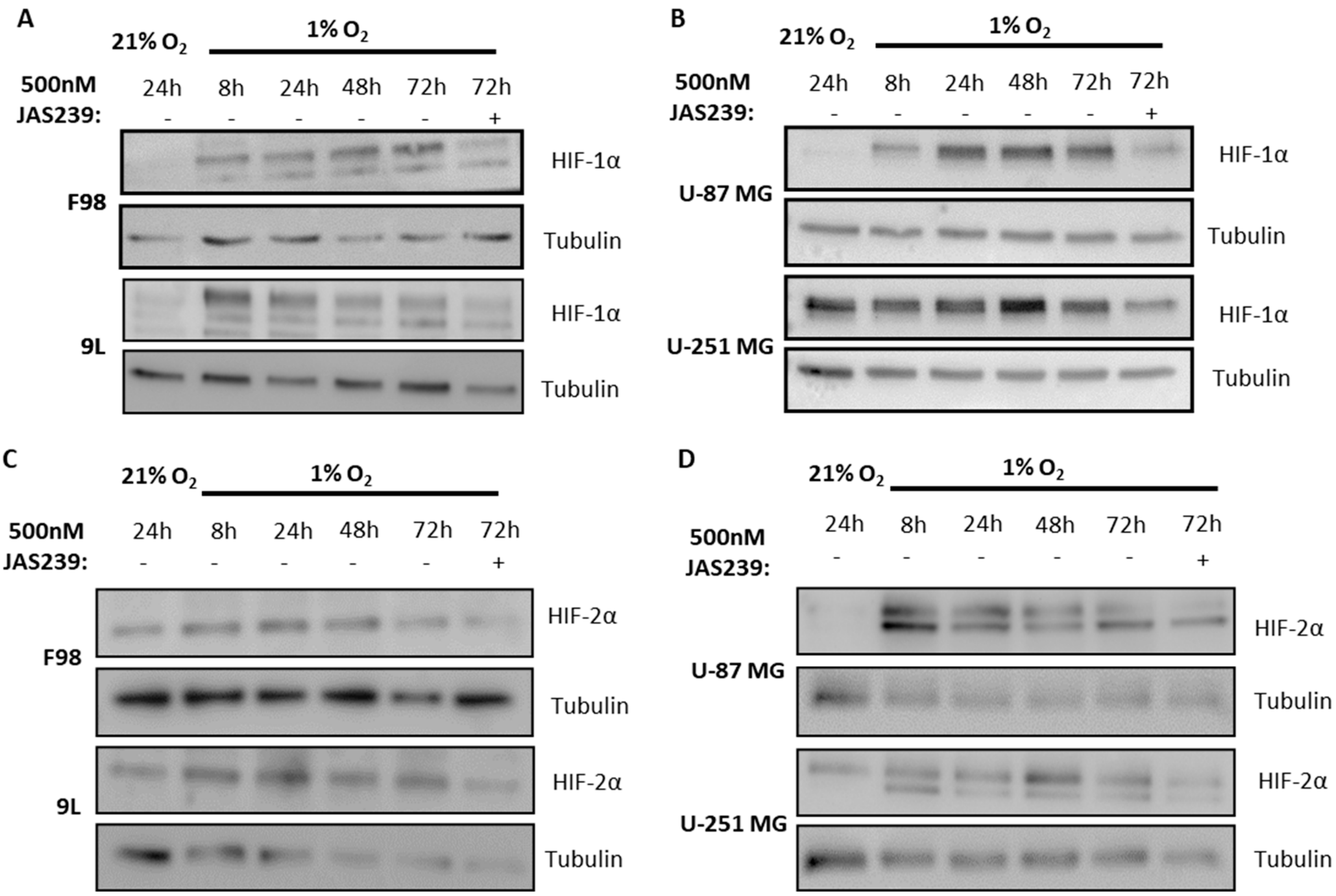

Figure S5. HIF-1α and HIF-2α protein expression was assessed over 72 hours and shows an induction in HIF-1α in response to hypoxia in all cell lines apart from U251 cells, where HIF-1α expression was already present in 21% O_2, and HIF-2α induction in all cell lines. 500nM JAS239 was added for 24 hours at the 72-hour time point. HIF-1α levels were reduced in all cell lines in presence of JAS239 (n=3).

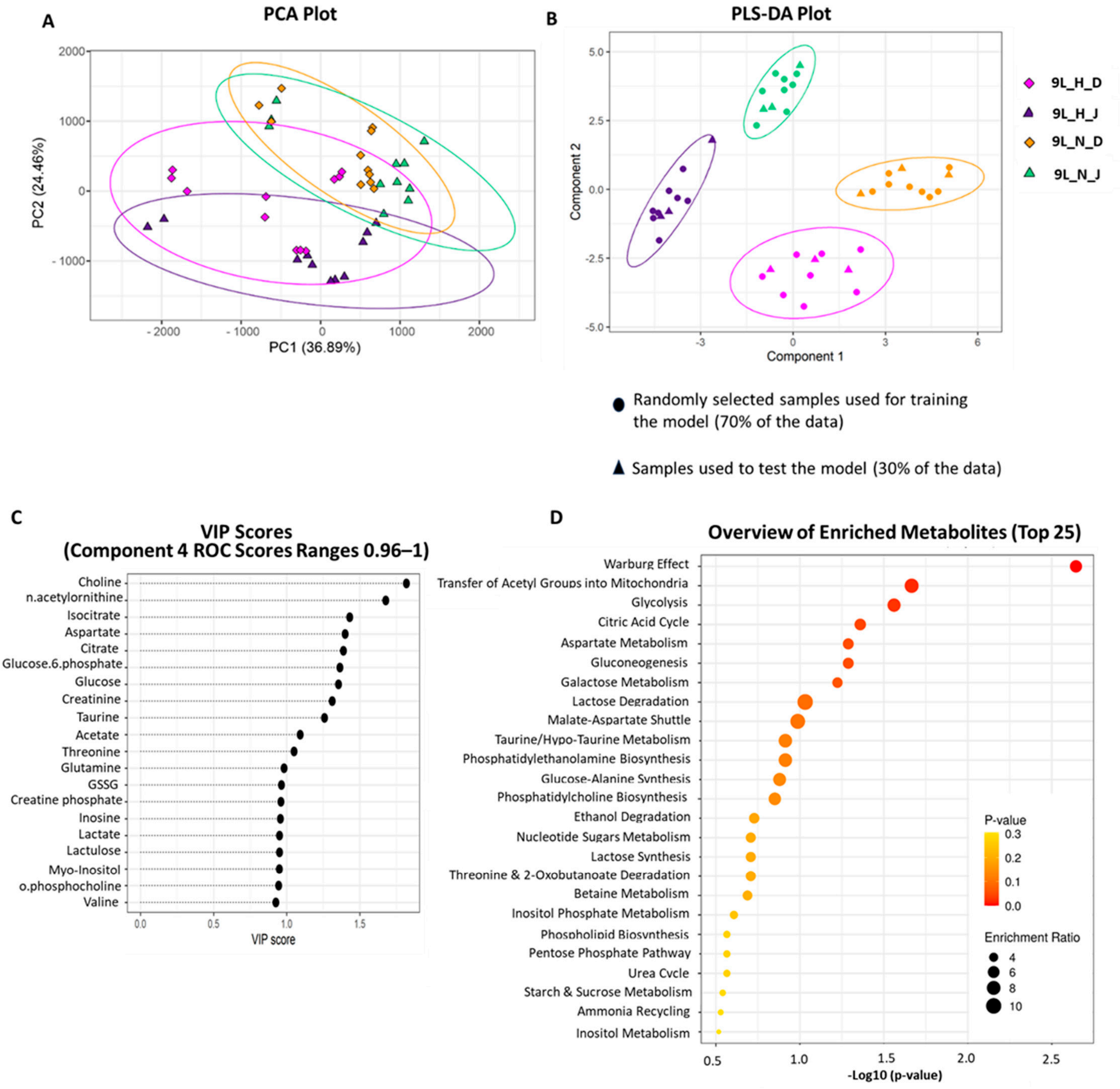

Figure S6. 9L metabolomic analysis pipeline. A) PCA analysis, B) PLS-DA analysis (component 4 ROC scores for each group vs all other groups: H_D = 1; H_J = 0.96; N_D = 1 and N_J = 0.96) and C) most influential metabolites. Metabolites with VIP >2 (C) informed the metabolite set enrichment analysis of top 20 metabolites using the hypergeometric test D). D, DMSO; H, hypoxia; J; JAS239; N, normoxia; PCA, principal component analysis; PLS-DA, partial least square discriminant analysis; ROC, receiver operating characteristic; VIP, variable importance in projection coefficients.

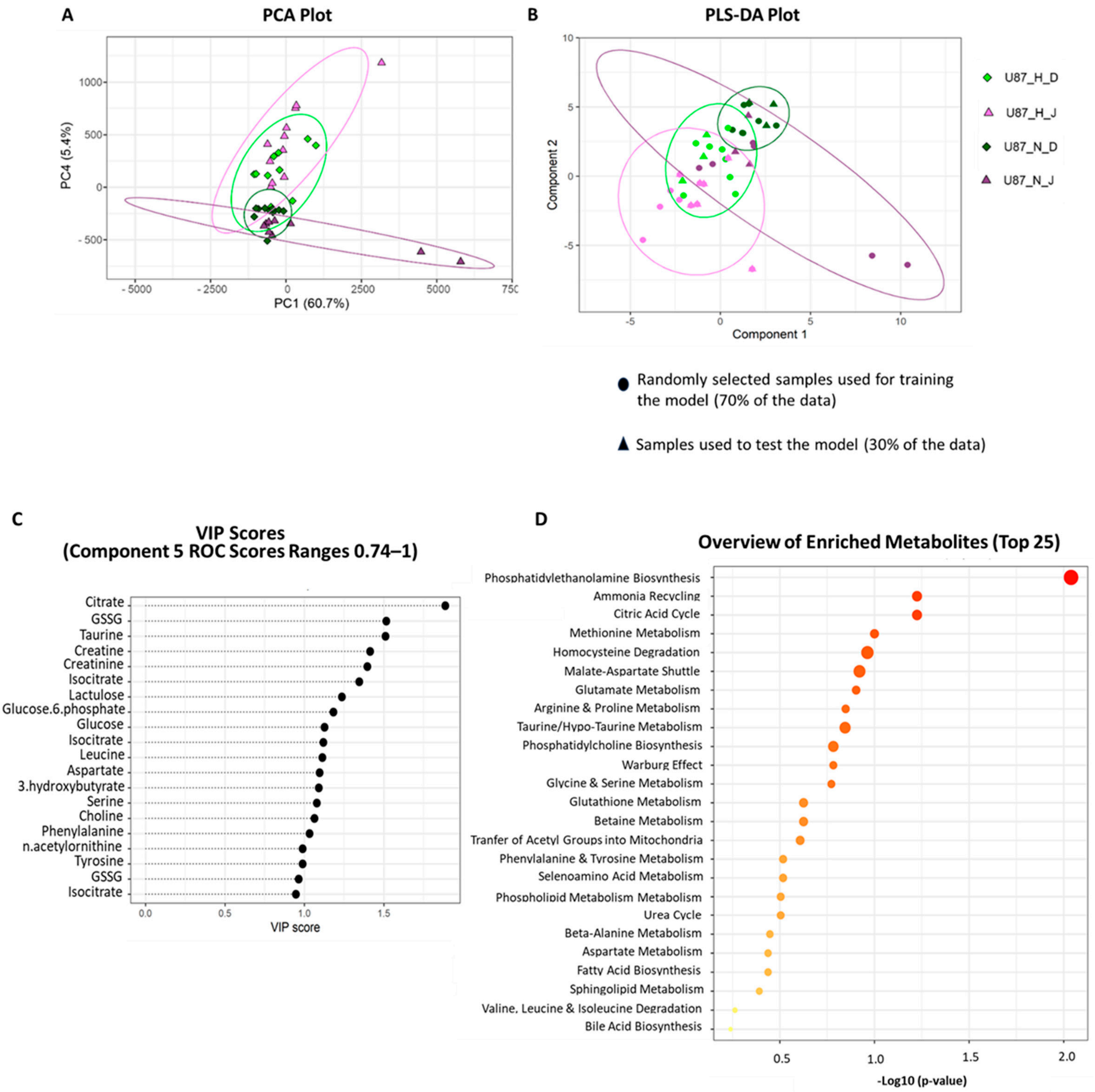

Figure S7. U-87 MG metabolomic analysis pipeline. A) PCA analysis, B) PLS-DA analysis (component 5 ROC scores for each group vs all other groups: H_D = 0.74; H_J = 1; N_D = 0.97 and N_J = 1) and C) most influential metabolites. Metabolites with VIP >2 (C) informed the metabolite set enrichment analysis of top 20 metabolites using the hypergeometric test D). D, DMSO; H, hypoxia; J; JAS239; N, normoxia; PCA, principal component analysis; PLS-DA, partial least square discriminant analysis; ROC, receiver operating characteristic; VIP, variable importance in projection coefficients.

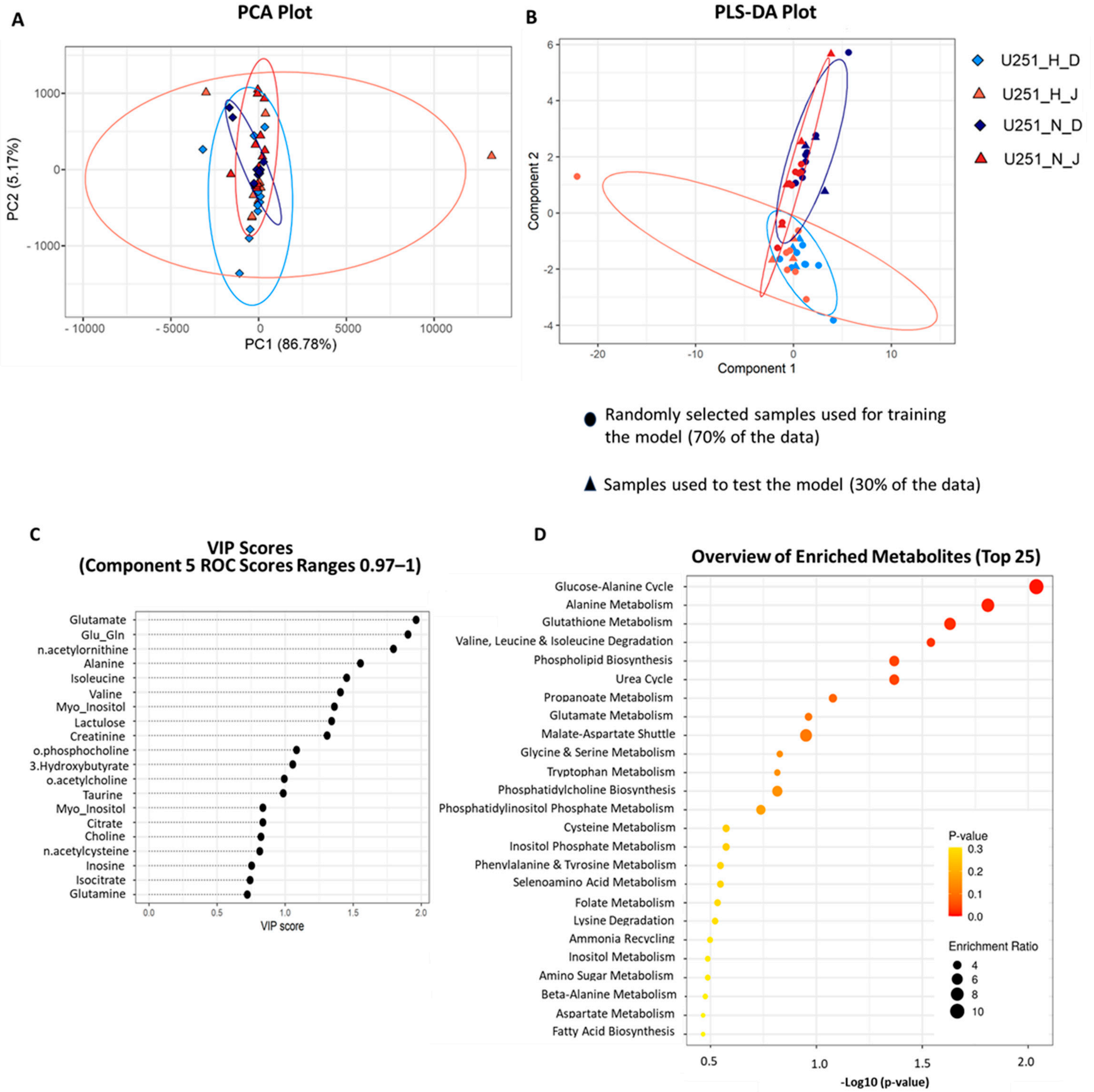

Figure S8. U-251 MG metabolomic analysis pipeline A) PCA analysis, B) PLS-DA analysis (component 5 ROC scores for each group vs all other groups: H_D = 0.97; H_J = 1; N_D = 1 and N_J = 1) and C) most influential metabolites. Metabolites with VIP >2 (C) informed the metabolite set enrichment analysis of top 20 metabolites using the hypergeometric test D). D, DMSO; H, hypoxia; J; JAS239; N, normoxia; PCA, principal component analysis; PLS-DA, partial least square discriminant analysis; ROC, receiver operating characteristic; VIP, variable importance in projection coefficients.

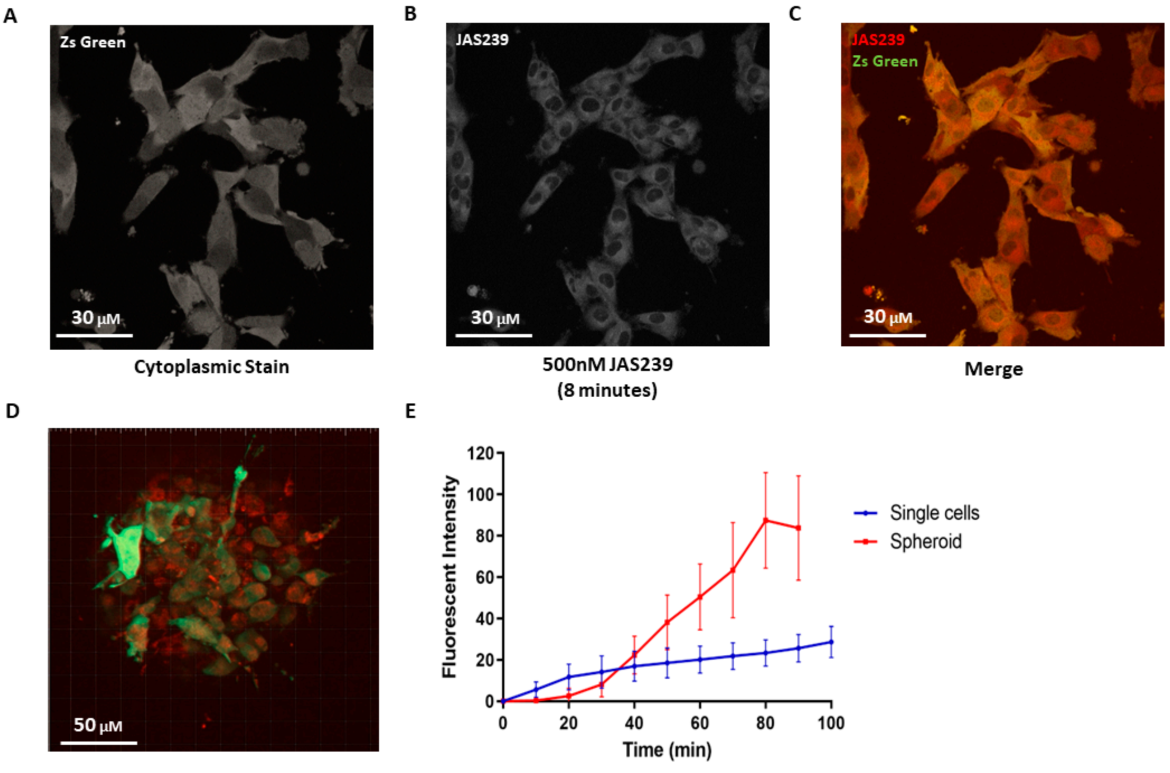

Figure S9. U-87 MG Zs Green labelled cells (green) were imaged on an Andor Dragonfly every 30 seconds for 2 hours in either a 2D (A–C) or 3D (D) model using 40x oil-based objective. 500nM JAS239 (red) was applied after the first z-stack was acquired. Image analysis was done using Imaris, after background subtraction, fluorescent intensity over time was plotted; FOV=3, n=3 (E).

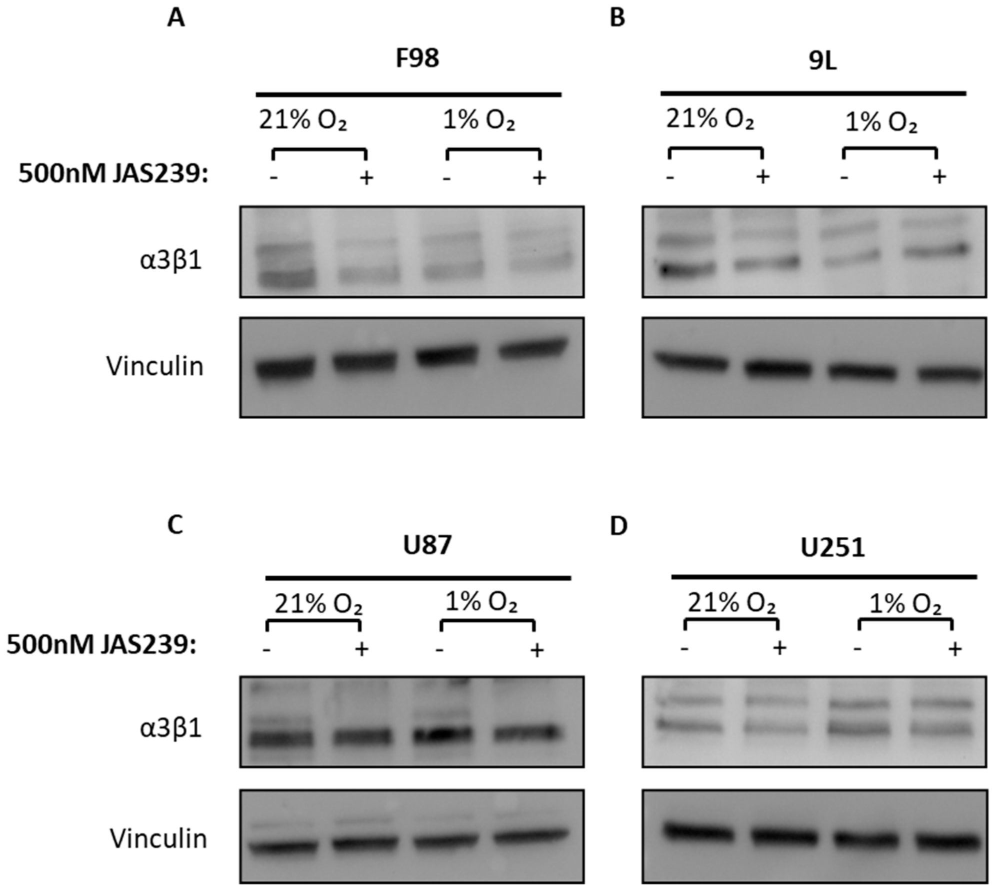

Figure S10. Cells were exposed to 21% or 1% O_2 for 96 hours before extraction. Protein expression of $\alpha3\beta1$ for all cell lines are visible A–D.
